# Screening of functional genes for hypoxia adaptation in Tibetan pigs by combined genome resequencing and transcriptome analysis

**DOI:** 10.3389/fvets.2024.1486258

**Published:** 2024-10-21

**Authors:** Bin Ni, Lin Tang, Li Zhu, Xinpeng Li, Kang Zhang, Hongyu Nie, Zeyu Ye, Yiwen Wang, Lijun Zhu, Xiaoyan Kong, Xiao Gou

**Affiliations:** ^1^School of Life Science and Engineering, Foshan University, Foshan, China; ^2^Faculty of Animal Science and Technology, Yunnan Agricultural University, Kunming, China

**Keywords:** Tibetan pig, resequencing, transcriptome, hypoxia adaption, functional gene

## Abstract

The high-altitude, low-oxygen environment of the Qinghai-Tibet Plateau poses significant challenges for the introduction of superior livestock breeds. However, local plateau species have adapted to thrive and reproduce under these harsh conditions. Understanding the molecular mechanisms behind plateau animals’ adaptation to low-oxygen environments is essential for breeding livestock suited to high-altitude regions. Tibetan pigs, which have undergone long-term natural selection and artificial breeding, have developed the ability to survive and reproduce in hypoxic environments. In this study, we conducted whole-genome resequencing of 30 Tibetan pigs from high-altitude regions and 30 Diannan small-ear pigs from low-altitude areas, to identify candidate genes that support Tibetan pigs’ adaptation to hypoxic conditions through selection signal analysis. Additionally, we performed transcriptome sequencing on five tissues (heart, liver, spleen, lung, and bone marrow) from both Tibetan pigs and Diannan small-ear pigs to identify genes with significant differential expression between the two breeds. We then integrated the genomic and transcriptomic data by examining the expression of candidate genes identified in selection signal analysis across different tissues. The selection signal analysis identified 10 genes—*HES*4, *ANGPT*1, *HIF3*A, *SPHK*2, *PCK*2, *RCN*3, *HIGD2*A, *DNM*2, *IRF*9, and *SRF*—that were under positive selection in the Tibetan pig population and are associated with hypoxia adaptation. When combined with transcriptome data, we found that five of these genes—*HIF3*A, *RCN*3, *HIGD2*A, *PCK*2, and *IRF*9—exhibited differential expression. Through an integrated approach of selection signal and transcriptome analysis, we identified five key functional genes that contribute to the adaptation of Tibetan pigs to hypoxic environments. These findings offer new insights into the adaptability of plateau animals.

## 1 Introduction

High-altitude regions are characterized by low temperatures, low oxygen levels, and intense ultraviolet (UV) radiation. These extreme environmental conditions pose significant challenges to the survival and adaptability of organisms, which limit the introduction of high-quality breeds. However, they have also driven the evolution of unique adaptive traits and reproductive capabilities in some species. By contributing to the sustainable development of animal husbandry, understanding the molecular mechanisms behind species’ adaptation to extreme environments is crucial for improving the resilience and productivity of other livestock breeds. This knowledge is also of great importance for the conservation and utilization of animal genetic resources (1–4). Compared to regular animals, high-altitude animals have stronger cardiopulmonary functions. They respond to hypoxia by increasing blood flow rate and oxygen content to supply tissues more efficiently (5, 6). Additionally, they exhibit higher red blood cell counts and hemoglobin levels, which enhance oxygen-binding and transport efficiency (7). The liver regulates iron metabolism by increasing EPO synthesis (8), while the spleen promotes stress-induced erythropoiesis and iron recycling (9). The bone marrow accelerates red blood cell production and regulates gene expression (10). These tissues and organs work together to ensure sufficient oxygen transport, enabling adaptation to low-oxygen environments. Tibetan pigs, in particular, are an ideal model for studying hypoxia adaptation in animals, as they have undergone extensive natural selection, making them highly adapted to low-oxygen environments (11).

Genomics and transcriptomics are commonly used techniques for identifying economic traits and breed characteristics in various species. In a genomic study, Shang Peng et al. (12) conducted selection signal analysis on ten geographically isolated Tibetan pig populations, revealing that genes such as *RXFP*1, *FZD*1, *OR*1*F*1, *TBX*19, *MSTN*, *ESR*1, *MC*1*R*, *HIF*3*A*, and *EGLN*2 are involved in lung development, coat color, and olfactory perception. On the transcriptomics side, Zhang et al. (13) used miRNA-seq technology to identify differentially expressed miRNAs (DE miRNAs) in the heart tissues of Tibetan and Yorkshire pigs raised at high altitudes. They discovered that the target genes regulated by these 20 DE miRNAs are enriched in signaling pathways related to hypoxia adaptation. However, past studies have often been limited to single-omics analyses, such as genomics focusing on DNA sequences and transcriptomics on RNA expression. While these studies provide biological insights at a certain level, their singular approach makes it difficult to fully understand the complexity of biological traits. In contrast, multi-omics integrated analysis can reveal the intricate molecular mechanisms of biological systems from multiple perspectives, thereby enhancing the comprehensiveness and reliability of research findings. Therefore, in this study, we combine genomics and transcriptomics to identify key functional genes involved in Tibetan pigs’ adaptation to hypoxic environments, with the goal of gaining a deeper understanding of their unique adaptive mechanisms.

In this study, we selected Tibetan pigs from high-altitude regions and Diannan small-ear pigs from low-altitude regions to conduct a genomic selection signal analysis. By comparing these two pig breeds from different altitudinal environments, our goal was to identify positively selected genes that contribute to the Tibetan pigs’ adaptation to low-oxygen conditions. Additionally, we collected heart, liver, spleen, lung, and bone marrow tissues from both pig breeds for transcriptome sequencing. Through the analysis of differentially expressed genes in these tissues, we aimed to identify key candidate genes involved in the environmental adaptation of Tibetan pigs. Subsequently, we performed an integrated analysis by comparing the positively selected genes identified in the genomic selection signal analysis of Tibetan pigs with the differentially expressed genes from the transcriptome analysis. This approach allowed us to pinpoint important functional genes responsible for the adaptation of Tibetan pigs to the hypoxic conditions of high-altitude environments. Ultimately, we identified five functional genes associated with this adaptation: *HIF*3A, *RCN*3, *HIGD*2*A*, *PCK*2, and *IRF*9. These findings provide a critical genetic foundation for understanding the mechanisms underlying the high-altitude adaptation of Tibetan pigs and offer valuable data resources and theoretical support for further research in this area.

## 2 Materials and methods

### 2.1 Sample collection

Thirty healthy Diannan small-ear pigs (DNSP) from Xishuangbanna, Yunnan Province, were selected for this study. Blood samples were collected from the ear vein into 5 mL collection tubes containing heparin sodium as an anticoagulant. The samples were stored and transported at −20°C and will be used for genomic DNA extraction. Additionally, the genomic resequencing data of 30 Tibetan pigs were downloaded from the NCBI database (Accession Number: PRJNA186497, Supplementary Table S1).

Moreover, three healthy Tibetan pigs (TP) from Diqing Tibetan Autonomous Prefecture (Altitude about 3,000 m) and three Diannan small-ear pigs (DNSP) from Xishuangbanna (Altitude about 500 m), Yunnan Province, were slaughtered. Heart, liver, spleen, lung, and bone marrow tissues were collected and placed into cryogenic vials pre-filled with RNA preservation solution. These samples were immediately stored in liquid nitrogen for subsequent RNA extraction and transcriptome sequencing. Animal protocol undergoing review and approval by the Experimental Animal Welfare and Animal Experiment Ethics Review Committee of Foshan University (approval number: FOSU202401-28). This process was conducted in accordance with the guidelines established by the Animal Use Committee of the Ministry of Agriculture of China, Beijing, aimed at minimizing animal suffering throughout the study.

### 2.2 Genomic analysis

#### 2.2.1 DNA extraction and whole-genome resequencing

Genomic DNA was extracted from the blood samples of 30 Diannan small-ear pigs using the TIANGEN Blood Genomic DNA Extraction Kit (DP348), following the manufacturer’s instructions. The quality and concentration of the extracted DNA were assessed using agarose gel electrophoresis and a UV spectrophotometer. Samples that passed quality control were sent to Beijing BerryGenomics Co., Ltd. for whole-genome resequencing on the DNBSEQ-T7 platform, with a sequencing depth of 30× for each sample.

#### 2.2.2 Detection of genetic variants

The raw sequencing data (raw reads) were first filtered and subjected to quality control using the Fastp (14) software with default parameters. The filtered clean reads were then aligned to the pig reference genome (version: Sscrofa11.1) using the “mem” command in the BWA (15) software. The resulting SAM files were converted to BAM format and sorted using the “sort” command in Samtools (16). PCR duplicates were identified and removed with the “markdup” command in the Sambamba (17) software. Subsequently, single nucleotide polymorphisms (SNPs) were identified using the “HaplotypeCaller” command in the GATK (18) software, and joint variant calling across multiple samples was performed with the “GenotypeGVCFs” command. Low-quality variant sites were filtered using the “VariantFiltration” command based on the following criteria: QD < 2.0 || MQ < 40.0 || FS > 60.0 || SOR > 3.0 || MQRankSum < −12.5 || ReadPosRankSum < −8.0. Finally, the Vcftools software was used with the options “--geno 0.1” and “--maf 0.01” to filter based on genotype missing rate and minor allele frequency, generating VCF files that record SNP sites for each sample for subsequent analysis.

#### 2.2.3 Selection signal analysis

Using the high-quality SNPs from the previous step, principal component analysis (PCA) was performed with the “pca” command in Plink (19) software to achieve data dimensionality reduction. The PCA results were visualized using R to check for any outlier samples. Next, nucleotide diversity (θπ) and the fixation index (Fst) between the two populations were calculated using the sliding window method in VCFtools (20), with a window size of 200 kb and a sliding step size of 100 kb (21). Regions ranked in the top 1% for both θπ and Fst were considered candidate regions under selection. These selected regions were then merged using Bedtools (22) software, and the genes within these regions were annotated based on the gene annotation file of the selected pig reference genome (Sus_scrofa.Sscrofa11.1.112.gtf). Finally, we used the KOBAS online tool to perform Gene Ontology (GO) and Kyoto Encyclopedia of Genes and Genomes (KEGG) enrichment analysis on the genes under positive selection identified in the Tibetan pig population.

### 2.3 Transcriptome analysis

#### 2.3.1 Tissue RNA extraction and transcriptome sequencing

Total RNA was extracted from the heart, liver, spleen, lung, and bone marrow tissues of Tibetan pigs and Diannan small-ear pigs using the TIANGEN RNA Easy Fast Animal Tissue Total RNA Extraction Kit (DP451), following the manufacturer’s instructions. The quality and concentration of the extracted RNA were assessed using agarose gel electrophoresis and a UV spectrophotometer. Samples that passed quality control were sent to Beijing BerryGenomics Co, Ltd. for transcriptome sequencing on the Illumina NovaSeq 6,000 platform.

#### 2.3.2 Identification of differentially expressed genes

The raw transcriptome sequencing data (raw reads) were filtered and subjected to quality control using Fastp software. The clean reads were then aligned to the pig reference genome (version: Sscrofa11.1) using hisat2 software. The resulting SAM files were converted to BAM format and sorted using the “sort” command in samtools. Subsequently, the “featureCounts” command in the subread software was used to count the number of reads mapped to each gene, and the results were normalized using the Transcripts Per Million (TPM) method. Principal component analysis (PCA) was then performed using the pcatools package in R to assess data quality and detect any outlier samples. Samples that passed quality control were subjected to differential expression analysis using the DESeq2 package, with screening criteria of |log2FoldChange| > 1 and Padj <0.05 to identify differentially expressed genes between the tissues.

### 2.4 Integrated analysis of selection signals and transcriptome data

An intersection analysis was conducted between the candidate genes identified as being under positive selection in the Tibetan pig population, which are likely involved in adaptation to hypoxic environments, and the differentially expressed genes identified across the five tissues. The intersecting genes, which are both under positive selection in the Tibetan pig population and differentially expressed between breeds, represent the results of the integrated analysis. By calculating Fst values and nucleotide diversity, combined with Tajima’s D, we can visualize the selection of genes in the Tibetan pig population. Tajima’s D is used to detect selection pressure; when one population has a Tajima’s D value below zero while the other is close to zero or positive, it may indicate positive selection in the former’s gene region. Combining Fst values with nucleotide diversity allows for more effective detection of genes under positive selection in the Tibetan pig population compared to Diannan small-ear pigs. The visualization of differentially expressed genes will use gene expression levels normalized by Transcripts Per Million (TPM) and will be presented through box plots.

### 2.5 Integrated analysis of selection signals and transcriptome data

To validate the results of the sequencing analysis, we performed RT-qPCR analysis on the selected genes. Total RNA was extracted from the corresponding high-expression tissue samples using TRIzol reagent (Invitrogen, Carlsbad, CA, United States), and then reverse transcribed into cDNA using the TIANGEN Kit KR210831. The 2 × SuperReal PreMix Plus (SYBR Green) was used for the qPCR reaction, with 2 μL of cDNA template and forward and reverse primers added. A reaction volume of 20 μL was used in the qPCR reactions according to the manufacturer’s protocol. qPCR experiments were performed in triplicate, and the average Ct was used for further analysis. The 2^−ΔΔCt^ method was used to determine relative mRNA abundance. The reaction conditions were as follows: 95°C for 15 min (1 cycle), 95°C for 10 s, and 60°C for 20 s (40 cycles). The sequences of the internal primers used for RT-qPCR are listed in Supplementary Table S4.

## 3 Results

### 3.1 Quality control and alignment of whole-genome resequencing data

The genome resequencing data of 30 Diannan small-eared pigs generated a total of 1.52 Tb of raw data. The whole-genome resequencing of 30 Diannan small-ear pigs generated a total of 1.52 Tb of raw data. After filtering and quality control, the average base quality scores for Q20 and Q30 were 96.22 and 90.33%, respectively. Q20 represents a base quality score where the probability of an incorrect base call is 1 in 100 (99% accuracy), while Q30 indicates a base quality score with a probability of 1 incorrect base call in 1,000 (99.9% accuracy). These metrics reflect the overall sequencing accuracy and the reliability of the data for downstream analysis. The average alignment rate was 99.43%, and the average sequencing depth was 19.33× (Supplementary Table S2). Similarly, the resequencing data of 30 Tibetan pigs downloaded from the NCBI database underwent the same processing, resulting in average base quality scores of 96.64% for Q20 and 90.80% for Q30, an average alignment rate of 99.61%, and an average sequencing depth of 5.81× (Supplementary Table S2). These results indicate that the quality of the whole-genome resequencing data is high and suitable for subsequent analyses.

### 3.2 Population selection signal analysis

**Figure 1:**
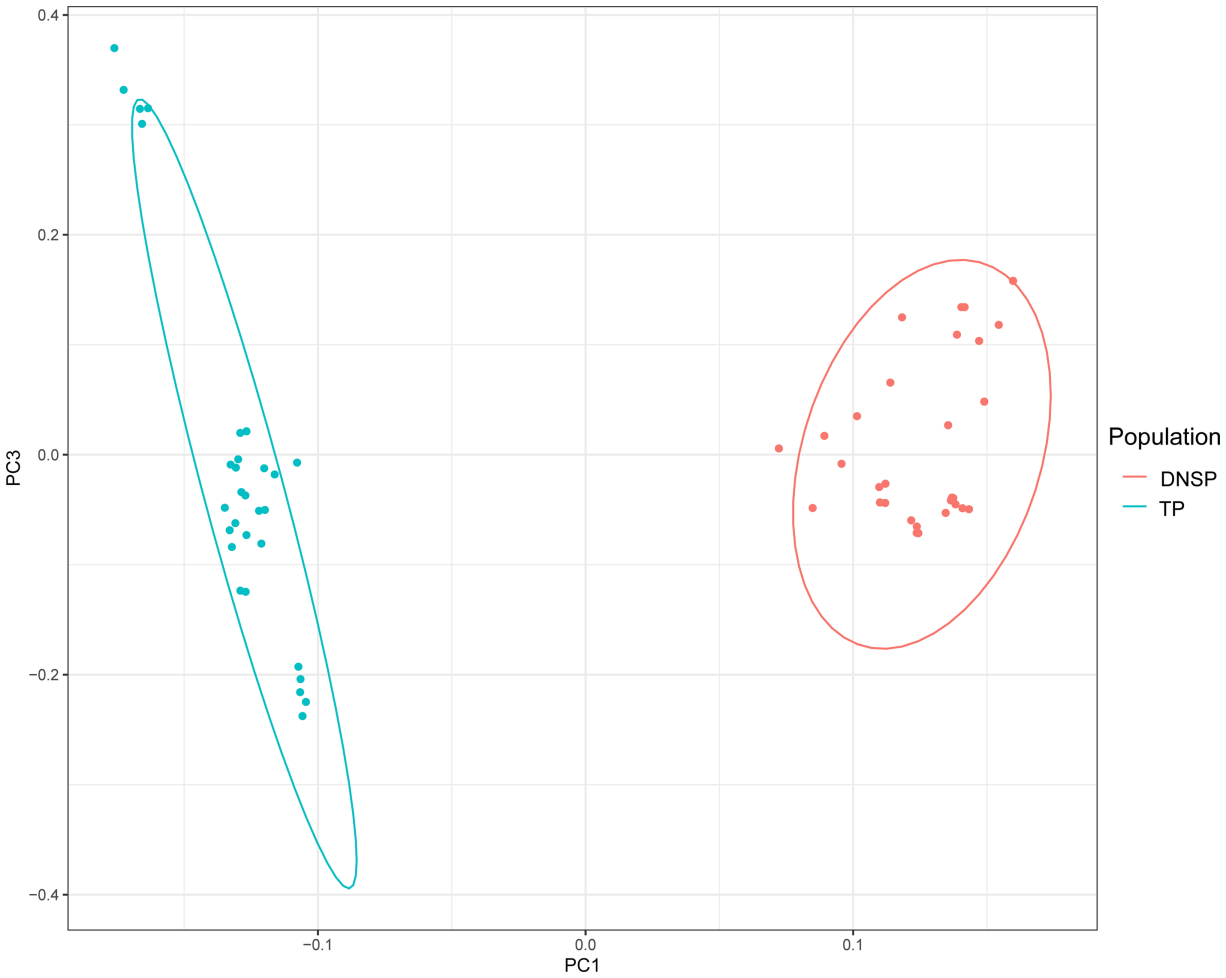
Genomic principal component analysis plot (PCA). Blue color represents the Tibetan pigs (TP) population and red color represents the Diannan small-ear pigs (DNSP) population.

The principal component analysis (PCA) results showed a clear separation between the two populations, with individuals from the same population clustering together. The PCA results were consistent with the expected classification (Figure 1). The genetic differentiation index (Fst) between the Tibetan pig and Diannan small-ear pig populations, calculated using the sliding window method, was 0.11. In population genetics, higher Fst values indicate greater genetic differentiation between two populations. An Fst value between 0.05 and 0.15 generally indicates moderate genetic differentiation. Therefore, both the PCA results and the Fst value suggest the presence of genetic differences between the two populations, supporting further exploration of these differences.

**Figure 2:**
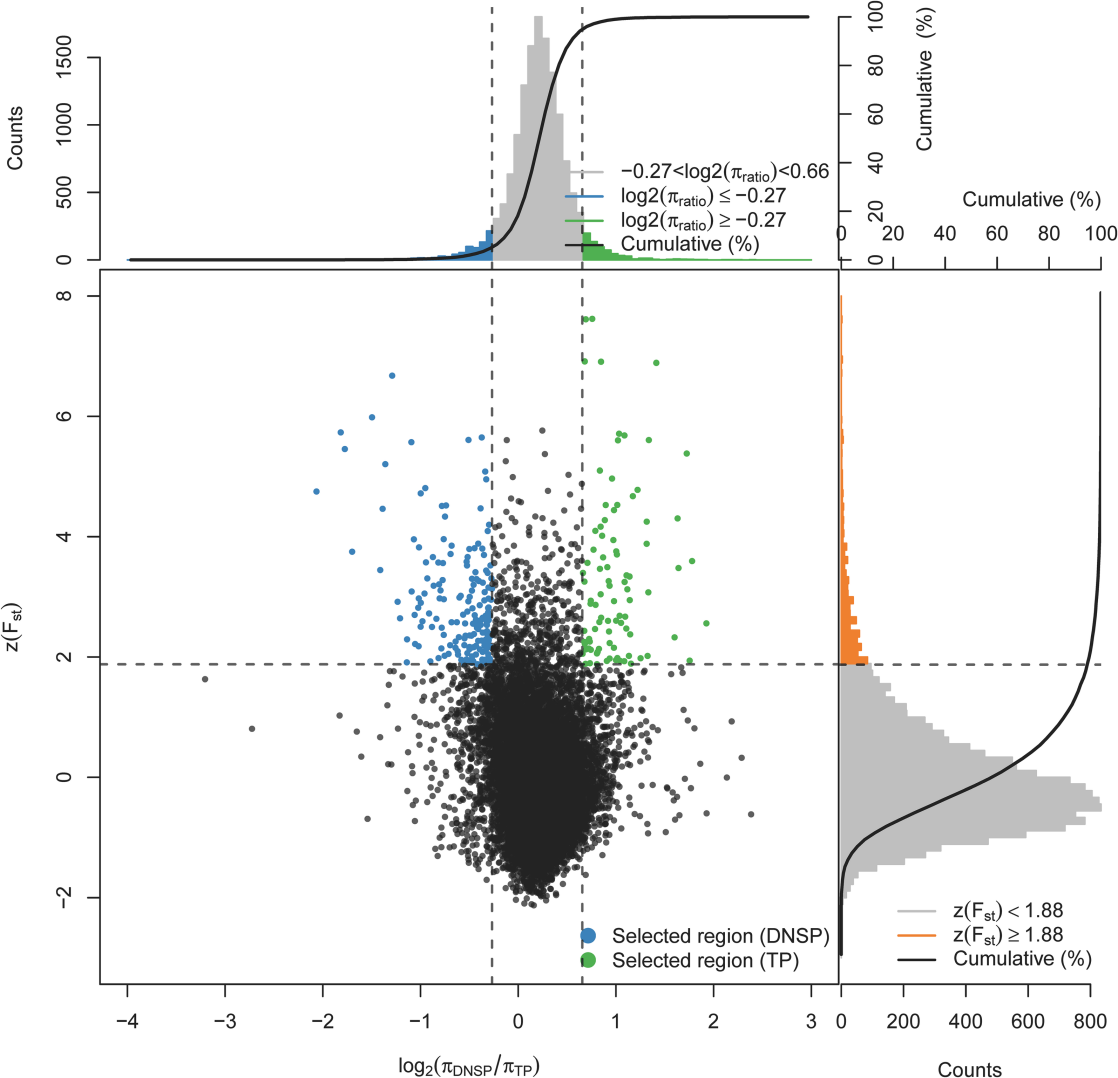
Distribution of Z(Fst) values and log2(π_TP/DNSP_) calculated in 200 kb sliding windows with 100 kb overlap between Tibetan pigs (TP) and Diannan small-ear pigs (DNSP). Green dots represent selected regions on the genome of TP, blue dots represent selected regions on the genome of DNSP.

To identify functional genes at the genomic level that may contribute to the adaptation of Tibetan pigs to hypoxic environments, we selected the Diannan small-ear pig, which lives in low-altitude areas, as the control group. A selection signal analysis was conducted using a combination of the genetic differentiation index Fst and nucleotide diversity θπ. The top 1% of regions were selected as candidate regions under selection, with thresholds of log2(θπ_DNSP_/θπ_TP_) ≥ −0.27 and Z(Fst) ≥ 1.88 (Figure 2). Gene annotation within the candidate regions revealed that 461 genes were under positive selection in the Tibetan pig population.

**Figure 3:**
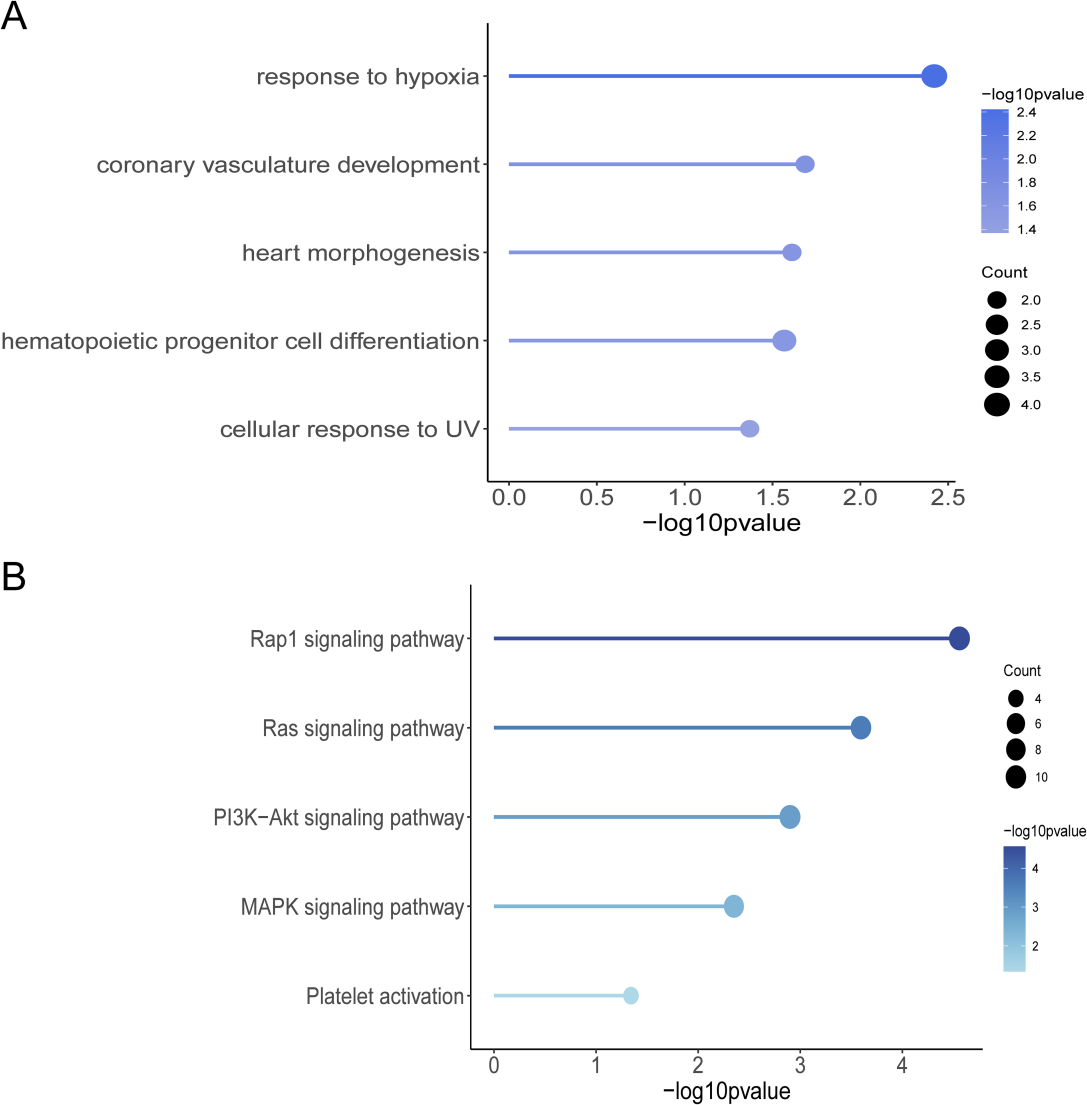
Selection of signaling screened genes for GO function and KEGG pathway enrichment analysis. **(A)** Graph of GO terms. **(B)** KEGG enrichment pathway diagram.

The results of the GO function and KEGG pathway enrichment analyses for the 461 genes. The GO enrichment analysis revealed significant enrichment of candidate genes in the following categories (Figure 3A): response to hypoxia, coronary vasculature development, heart morphogenesis, hematopoietic progenitor cell differentiation, and cellular response to UV. These genes may contribute to the adaptation of Tibetan pigs to the hypoxic and high UV environments of high-altitude regions by influencing vascular development, hematopoietic function, and cellular responses to ultraviolet radiation (23–25). In the KEGG pathway enrichment analysis, the candidate genes were predominantly enriched in the following pathways (Figure 3B): Rap1 signaling pathway, Ras signaling pathway, PI3K-Akt signaling pathway, MAPK signaling pathway, and platelet activation pathway. These pathways are primarily involved in vascular formation and repair, suggesting that angiogenesis is a key process in the adaptation of Tibetan pigs to hypoxic conditions (13, 26, 27). By examining the functions of genes associated with hypoxia adaptation within the enriched GO terms and KEGG pathways, and integrating analyses of Fst, nucleotide diversity, and Tajima’s D, we identified 10 candidate genes: *HES*4, *ANGPT*1, *HIF*3*A*, *SPHK*2, *PCK*2, *RCN*3, *HIGD*2*A*, *DNM*2, *IRF*9, and *SRF*. Selection signal analysis indicates that these genes have undergone positive selection in the Tibetan pig population, as evidenced by significantly high Fst values within the selected regions, reduced nucleotide diversity in the selected population, and notable differences in Tajima’s D values between the two populations (Supplementary Figure S1).

### 3.3 Quality control and alignment of transcriptome sequencing data

RNA from five tissues (heart, liver, spleen, lung, and bone marrow) of both Tibetan pigs and Diannan small-ear pigs was collected for transcriptome sequencing, with three biological replicates per group, resulting in a total of 30 transcriptome sequencing datasets. After filtering and aligning low-quality bases, the average base quality scores of Q20 and Q30 were 97.23 and 94.85%, respectively, and the average alignment rate was 99.23% (Supplementary Table S3). These results indicate that the transcriptome sequencing data are reliable.

### 3.4 Identification of differentially expressed genes

**Figure 4:**
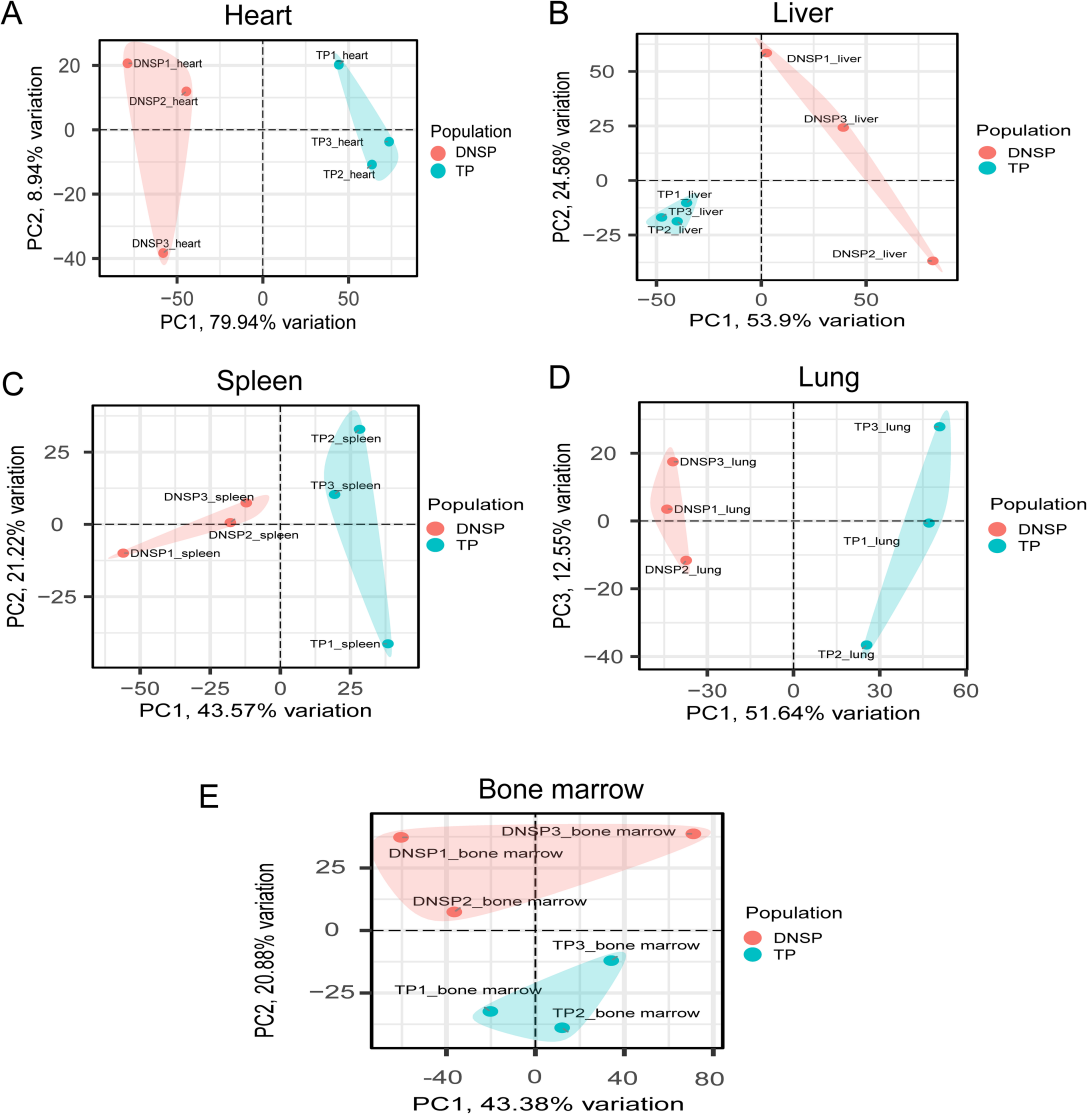
PCA plots of five tissues of Tibetan pigs (TP) and Diannan small-ear pigs (DNSP). **(A)** Heart tissue PCA **(B)** Liver tissue PCA **(C)** Spleen tissue PCA **(D)** Lung tissue PCA **(E)** Bone marrow tissue PCA.

**Figure 5:**
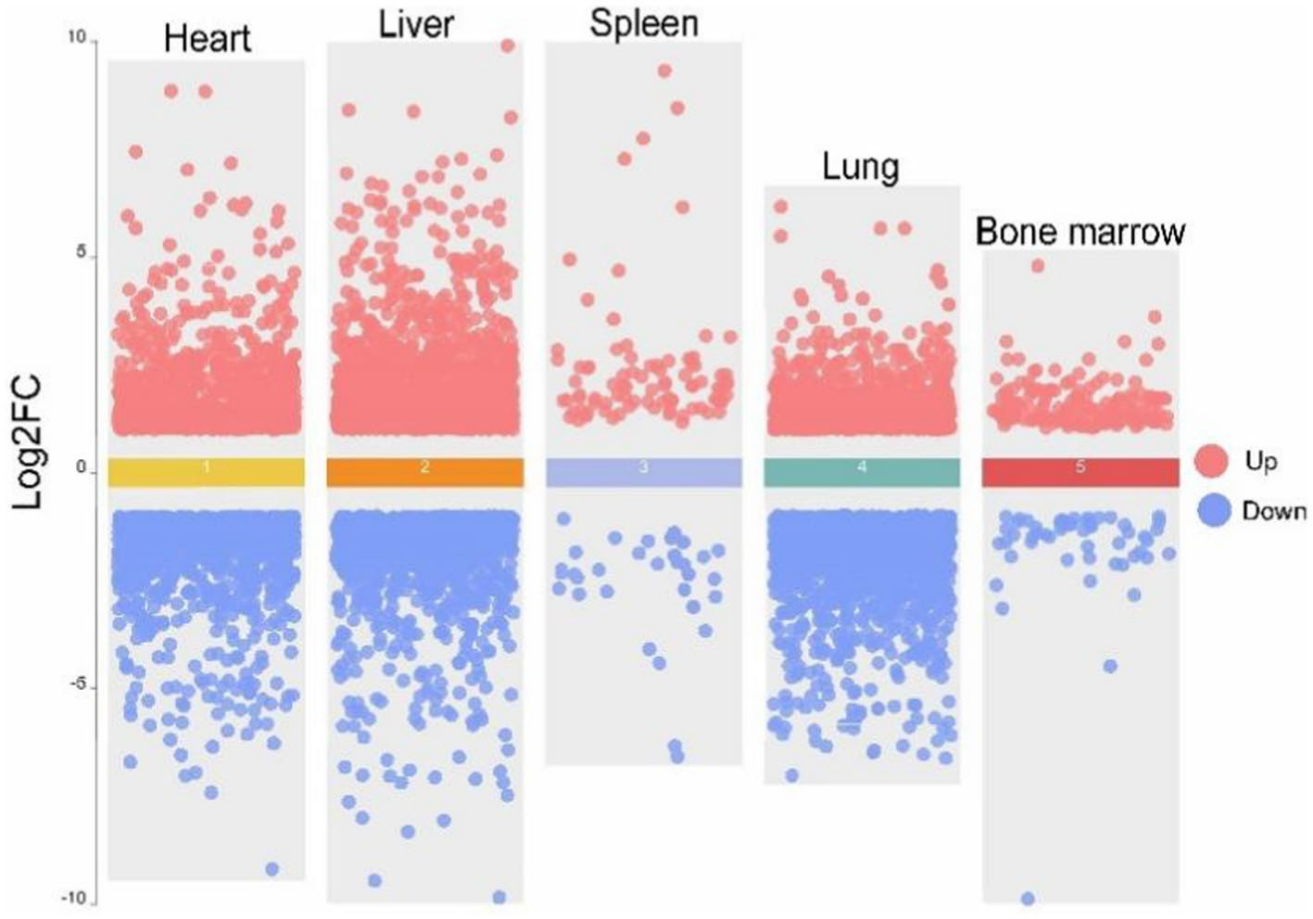
Volcano map of five tissues of heart, liver, spleen, lung and bone marrow of Tibetan pigs (TP) and Diannan small-ear pigs (DNSP). Red color indicates up-regulation of differentially expressed genes and blue color indicates down-regulation of differentially expressed genes.

To ensure the reliability of the differential expression analysis, we first performed principal component analysis (PCA) on the TPM-normalized gene expression matrix using the PCAtools package in R to detect any outlier samples. The results (Figure 4) showed that tissue samples from the same breed exhibited good clustering across different tissues, with no obvious outliers, indicating high sequencing quality. Subsequently, we used the DESeq2 package to analyze differentially expressed genes (DEGs), with the criteria for selection being |log2Fold Change| > 1 and Padj <0.05. The results of the differential expression analysis for the various tissues are as follows (Figure 5). In heart tissue, we identified 1,762 DEGs (918 upregulated and 844 downregulated). In liver tissue, 2,437 DEGs were identified (1,328 upregulated and 1,109 downregulated). In spleen tissue, 119 DEGs were identified (86 upregulated and 33 downregulated). In lung tissue, 2,504 DEGs were identified (1,070 upregulated and 1,434 downregulated). Finally, in bone marrow tissue, 212 DEGs were identified (159 upregulated and 53 downregulated).

### 3.5 Integrated analysis of selection signals and transcriptome data

**Figure 6:**
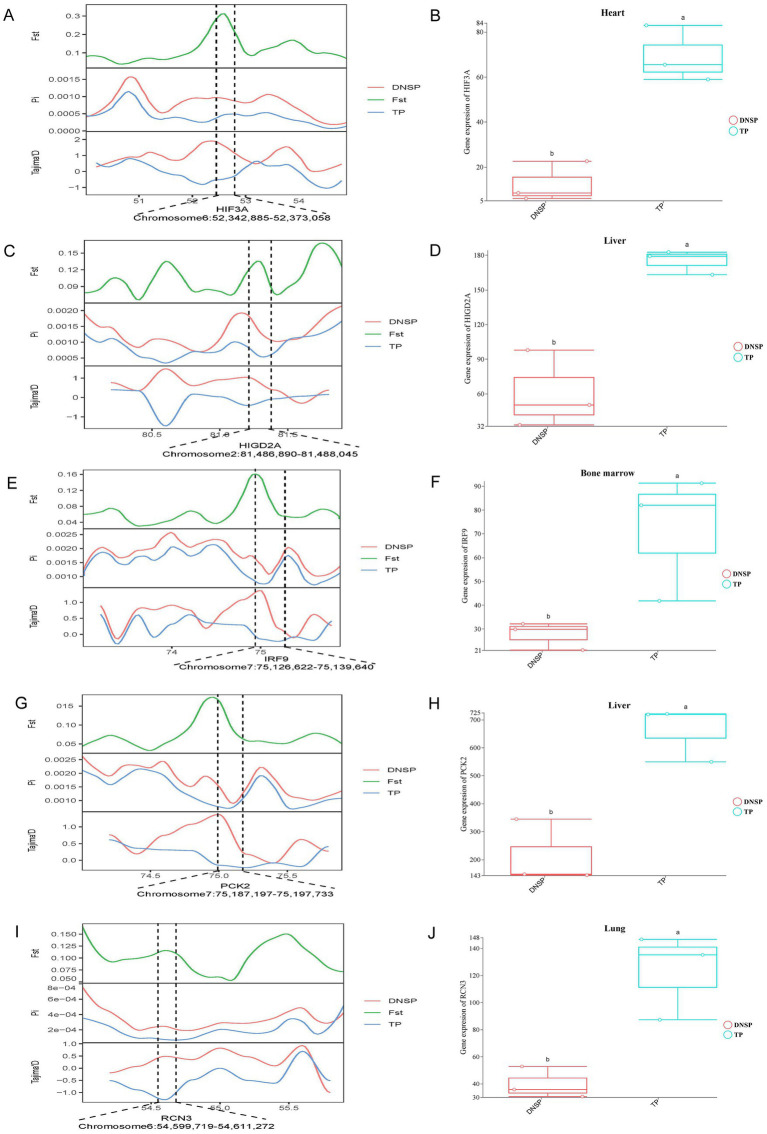
Combined analysis of genomic selection signal candidate genes and transcriptome differentially expressed genes in Tibetan pigs (TP) and Diannan small-ear pigs (DNSP). **(A)** Fst, Pi and Tajima’s D value of *HIF*3*A* between TP and DNSP. **(B)** Differentially expressed genes in heart tissue of TP and DNSP. **(C)** Fst, Pi and Tajima’s D value of *HIGD*2*A* between TP and DNSP. **(D)** Differentially expressed genes in Liver tissue of TP and DNSP. **(E)** Fst, Pi and Tajima’s D value of *IRF*9 between TP and DNSP. **(F)** Differentially expressed genes in Bone marrow tissue of TP and DNSP. **(G)** Fst, Pi and Tajima’s D value of *PCK*2 between TP and DNSP. **(H)** Differentially expressed genes in Liver tissue of TP and DNSP. **(I)** Fst, Pi and Tajima’s D value of *RCN*3 between TP and DNSP. **(J)** Differentially expressed genes in Lung tissue of TP and DNSP.

In the selection signal analysis, we identified 10 candidate genes—*HES*4, *ANGPT*1, *HIF*3*A*, *SPHK*2, *PCK*2, *RCN*3, *HIGD*2*A*, *DNM*2, *IRF*9, and *SRF*—through functional annotation of genes under selection in the Tibetan pig population and a review of relevant literature (12, 28–36). By examining the expression of these 10 genes across five tissues in the two populations, we found that *HIF*3*A* showed significant expression in heart tissue (Figures 6A,B), *HIGD*2*A* and *PCK*2exhibited significant differential expression in liver tissue (Figures 6C,D,G,H), *IRF*9 showed significant differential expression in bone marrow (Figures 6E,F), and *RCN*3 exhibited significant expression in lung tissue (Figures 6I,J). The results from the selection signal analysis show that the regions in the genome under selection exhibit high Fst values. In population genetics studies, it is generally considered that when Fst values range between 0.05 and 0.15, there is moderate genetic differentiation between populations. In this joint analysis, the Fst values of the five selected genes were all greater than 0.05, indicating moderate genetic differentiation in the regions where these genes are located. When populations undergo positive selection, the selected population typically displays lower nucleotide diversity, and the Tajima’s D values between populations differ, with the Tajima’s D of the selected population usually being less than 0. In the regions where these genes are located, the nucleotide diversity of the Tibetan pig population is lower than that of the Diannan small-ear pig, and its Tajima’s D value is not only lower than that of the Diannan small-ear pig population but also less than 0. This indicates that these five genes are under positive selection in the Tibetan pig population. Combined with the results of transcriptome analysis, we found that there were also significant differences in the expression of these five genes in the tissues. Therefore, *HIF*3*A*, *RCN*3, *HIGD*2*A*, *PCK*2, and *IRF*9 have been identified as key functional genes associated with hypoxia adaptation through the integrated analysis of selection signals and transcriptome data.

### 3.6 Quantitative real-time PCR (qPCR)

**Figure 7:**
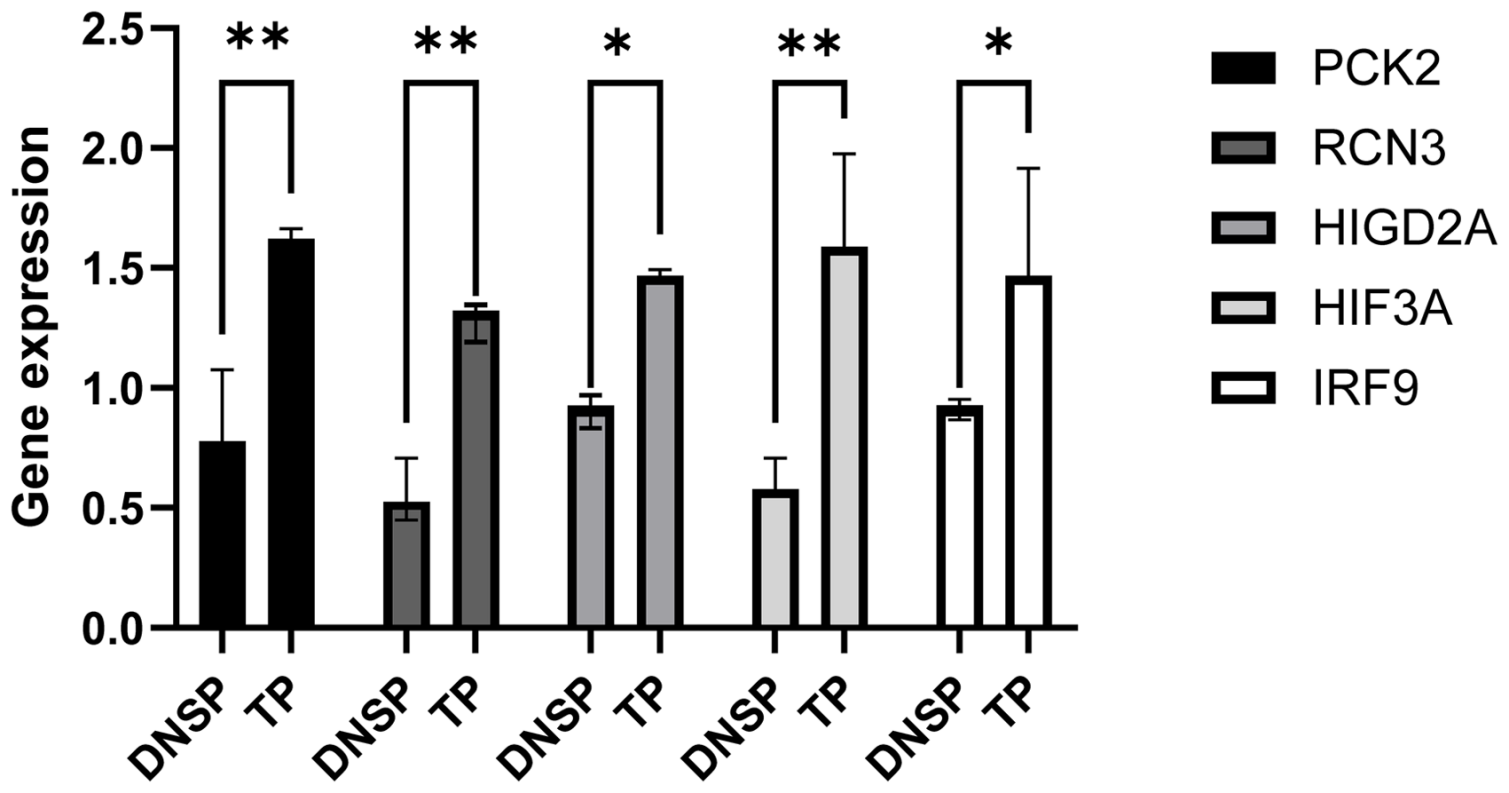
Validation of gene expression patterns obtained from RNA-seq data. qPCR was used to validate the differential expression of the *HIF*3*A* gene in heart tissue, the *HIGD*2*A* and *PCK*2 genes in liver tissue, and the *RCN*3 gene in lung tissue.

We validated the RNA-seq data for the five selected genes, *HIGD*2*A*, *HIF*3*A*, *RCN*3, *PCK*2, and *IRF*9, and found significantly higher expression levels in Tibetan pigs living in high-altitude areas compared to Diannan small-ear pigs from lower altitudes (Figure 7). These results suggest that identifying DEGs through transcriptome data is effective and reliable.

## 4 Discussion

### 4.1 Genetic differences between Tibetan pigs and Diannan small-ear pigs

Archaeological and genetic data provide significant evidence that human migration and cultural expansion during the late Pleistocene (22,000–18,000 years ago) and the early Neolithic period (10,000–7,000 years ago) influenced the ecological and agricultural development of the plateau regions. This process introduced agriculture to the Himalayas and facilitated the establishment of animal husbandry on the plateau, impacting the domestication and gene flow of high-altitude animals (37–39). The origin of Tibetan pigs is closely linked to the migration of the Tibetan people, as evidenced by 5,000-year-old pig bones from the Karuo archaeological site, which suggest that the Tibetans domesticated Tibetan pigs (11). Due to their long-term adaptation to the plateau environment, Tibetan pigs have evolved unique physiological traits, such as a small body size, long hair, and thick subcutaneous fat, which help them withstand the cold climate. Additionally, Tibetan pigs have a higher red blood cell count, increased hemoglobin levels, and more efficient metabolism, allowing them to utilize oxygen more effectively and survive at high altitudes (40–42). In contrast, Diannan small-ear pigs primarily inhabit the subtropical regions of southern Yunnan Province and exhibit genetic adaptations to the hot and humid climate of tropical and subtropical environments. These adaptations include smaller ears, a slightly longer snout, and a short, thick neck, enabling them to thrive in low-altitude, humid conditions (43–45). Overall, the significant differences in environmental adaptability and biological characteristics between Tibetan pigs and Diannan small-ear pigs make them ideal models for studying the adaptability and traits of pig breeds in different environments. In this study, we first used principal component analysis (PCA) and the genetic differentiation index (Fst) to investigate the two populations. The PCA results revealed clear genetic differences between the two populations, while the Fst value indicated a moderate level of genetic differentiation between them, consistent with our expectations.

### 4.2 Analysis of genomic selection signals

In this study, 461 candidate genes were identified through genomic selection signal analysis. After conducting KEGG pathway and GO functional enrichment analyses, and reviewing relevant literature, 10 potential hypoxia adaptation genes were identified: *HES*4, *ANGPT*1, *HIF*3*A*, *SPHK*2, *PCK*2, *RCN*3, *HIGD*2*A*, *DNM*2, *IRF*9, and *SRF*. Chen et al. (28) performed single-cell transcriptome analysis on yak heart tissue and, through cell type annotation and differential expression analysis, found that altitude changes primarily affected the expression patterns of smooth muscle cells and vascular endothelial cells. Four key transcription factors (*MEF*2*B*, *FOXP*4, *ARID*5*A*, and *HES*4) were identified in smooth muscle cells. Among them, *HES*4 can help cells cope with environmental stress, such as hypoxia, by regulating the expression of heat shock proteins (*HSPs*). Therefore, *HES*4 may be a potential gene for high-altitude hypoxia adaptation in Tibetan pigs. The *ANGPT*1 gene, a crucial angiogenic factor, can promote tissue angiogenesis and enhance oxygen supply (46). It has been identified as a positively selected gene in the Tibetan population (47). Previous research has also identified *ANGPT*1 as a candidate gene for high-altitude adaptation by comparing the whole-genome sequences of wolves from different altitudes (29). *HIF*3*A* has been reported to promote high-altitude adaptation in both Tibetan pigs (12) and yaks (48), although some studies suggest it is also associated with obesity (49). Hypoxia activates hypoxia-inducible factors (*HIFs*), leading to changes in the expression of downstream genes. *HIF-*3α can directly bind to erythropoietin (*EPO*) and induce its transcription (50). *EPO* is a key growth factor that regulates red blood cell count and hematocrit, and is controlled by hypoxia-inducible factor-2α (*HIF*-2*α*). It is produced in the liver during early development and later in the kidneys (51), playing a crucial role in hypoxia adaptation (52–54). In mouse kidney fibroblast cell lines, knockdown of the sphingosine kinase 2 (*SPHK*2) gene was found to increase the synthesis of *HIF*-2*α* and *EPO* (55). Other studies have shown that *SPHK*2 is associated with hypoxia-inducible factor-1α (*HIF*-1*α*) within protein complexes, and is enriched in the promoter regions of HIF target genes such as vascular endothelial growth factor (*VEGF*), enhancing local histone H3 acetylation and transcription (30). Under various internal and external stress factors such as hypoxia, glucose deprivation, ATP depletion, calcium overload, and reduced protein degradation, the endoplasmic reticulum can lose its homeostasis (56). Phosphoenolpyruvate carboxykinase 2 (*PCK*2) is involved in cellular metabolism, including glucose metabolism and the tricarboxylic acid cycle (TCA), and can promote endoplasmic reticulum stress, playing a vital role in maintaining cellular homeostasis and activity (57). *RCN*3, a calcium-binding protein, plays a role in calcium homeostasis and protein folding within the cell. Calcium ions have multiple functions in cardiac and pulmonary tissues, including promoting myocardial contraction and relaxation, maintaining heart rhythm, regulating alveolar ventilation and gas exchange, and participating in cell signaling and metabolic regulation. Calcium ion homeostasis is crucial for the normal function of cardiac and pulmonary tissues (58, 59). *HIGD*2*A*, which encodes a hypoxia-induced domain protein, is involved in the assembly of the mitochondrial respiratory chain and is essential for cell survival under hypoxic conditions (33). *DNM*2 is a key regulator of intracellular membrane dynamics, including endocytosis and vesicle transport. Research by Joshi et al. has found a regulatory relationship between *DNM*2 and hypoxia-inducible factor-1α (*HIF*-1*α*) (34). Interferon regulatory factor 9 (*IRF*9) is an important transcription factor in the interferon signaling pathway. Studies have shown that interferon signaling can regulate the expression or activity of *HIF*-1*α*, thereby influencing cellular adaptation to hypoxia. Conversely, *HIF*-1*α* can regulate some interferon-related genes, affecting immune responses (60, 61). Serum response factor (*SRF*) plays a critical role in regulating cell growth, differentiation, and survival. Research has shown that *SRF* is important in hypoxia adaptation, as it can regulate the expression of various genes related to the hypoxic response (36, 62, 63). In summary, through selection signal analysis, we have identified 10 key candidate genes potentially involved in the hypoxia adaptation of Tibetan pigs.

### 4.3 Combined analysis of selection signals and transcriptomics

Gene expression regulation plays a crucial role in species adaptation to environmental changes. This complex biological process allows organisms to respond to and adapt to various environmental shifts by modulating the activity of specific genes (64). Therefore, in this study, we conducted transcriptomic analysis on five tissues—heart, liver, spleen, lung, and bone marrow—from Tibetan pigs and Diannan small-ear pigs. These tissues encompass key functions such as blood circulation, oxygen transport, and hematopoiesis, enabling us to identify important functional genes involved in hypoxia adaptation in Tibetan pigs through differential gene expression analysis. After identifying differentially expressed genes across various tissues, we conducted a combined analysis of the 10 candidate genes for hypoxia adaptation, previously identified through selection signal screening, and the differentially expressed genes. The results revealed that five genes—*HIF*3*A*, *RCN*3, *HIGD*2*A*, *PCK*2, and *IRF*9—not only underwent positive selection in the Tibetan pig population but also showed significant differential expression in various tissues. Animals living in high-altitude regions often exhibit enhanced cardiopulmonary function to cope with hypoxic environments by increasing blood flow rate and oxygen content (5, 6, 65). We found that the *HIF*3*A* gene was differentially expressed in heart tissue. As a member of the HIF family, *HIF*3*A*, along with other members like *HIF*1*A*, can promote angiogenesis under hypoxic conditions (66). The high expression of *HIF*3*A* in heart tissue may enhance the formation of blood vessels within the heart, thereby improving its blood supply capacity. The *RCN*3 gene was differentially expressed in lung tissue. Hypoxic environments can trigger endoplasmic reticulum stress in cells, potentially leading to protein misfolding (67). As an endoplasmic reticulum calcium-binding protein, the high expression of RCN3 in the lungs of Tibetan pigs may help enhance the cell’s ability to cope with hypoxia-induced endoplasmic reticulum stress, thus protecting lung cells from hypoxic damage. *PCK*2 and *HIGD*2*A* were both highly expressed in the liver tissue of Tibetan pigs. *PCK*2 encodes an enzyme involved in gluconeogenesis, and its high expression may enhance this pathway, helping to maintain blood glucose levels and ensuring sufficient energy supply to glucose-dependent tissues such as the brain (31). *HIGD*2*A* is involved in regulating mitochondrial function, and its high expression may help maintain mitochondrial respiratory efficiency in the liver, ensuring energy balance under hypoxic conditions (33, 68). The *IRF*9 gene was highly expressed in the bone marrow tissue of Tibetan pigs. IRF9 is a key regulator in the interferon signaling pathway, playing a role in the immune response. The *IRF* gene family is also important in the differentiation of hematopoietic cells (69). While *IRF*9 is primarily considered an immune regulator, its potential role in hematopoiesis should not be overlooked. In summary, through combined analysis, we identified five key genes that contribute to the hypoxia adaptation of Tibetan pigs.

In this study, although several important candidate genes related to the high-altitude adaptation of Tibetan pigs were identified through genomic selection signal analysis and transcriptomic analysis, there are still some limitations that need to be considered. This research primarily focused on two specific pig populations—Tibetan pigs and Diannan small-ear pigs. While these pig breeds exhibit significant differences in adapting to different environments, the differences in their genomes and transcriptomes may not represent the adaptive evolution of all high-altitude and low-altitude pig breeds. Therefore, the generalizability of the research findings requires further validation. This study experimental validation was limited to qPCR, which somewhat restricts the in-depth understanding of the functions of these genes. While qPCR can verify the expression differences of candidate genes in various tissues, this method does not directly reveal the specific functional mechanisms of these genes in low-oxygen adaptation. Therefore, future research should adopt additional experimental methods for functional validation to enhance the depth and reliability of the study, thereby providing a comprehensive understanding of the molecular mechanisms underlying Tibetan pigs’ adaptation to the low-oxygen environment of high altitudes.

## 5 Conclusion

Based on the analysis of whole-genome resequencing data, we annotated genes within candidate regions and identified 461 genes under positive selection in the Tibetan pig population. Through a combined analysis of selection signals and transcriptomic data, we ultimately identified five key functional genes—*HIF*3*A*, *RCN*3, *HIGD*2*A*, *PCK*2, and *IRF*9—that contribute to the adaptation of Tibetan pigs to hypoxic environments. These findings offer new insights into the molecular mechanisms underlying hypoxia adaptation in high-altitude animals and provide potential molecular markers for future breeding strategies.

## Data Availability

The datasets presented in this study can be found in online repositories. The names of the repository/repositories and accession number(s) can be found at: https://www.ncbi.nlm.nih.gov/, PRJNA1164910, PRJNA1148337.

## References

[1] Tong C Tian F Tang Y Feng C Guan L Zhang C. Positive Darwinian selection within interferon regulatory factor genes of *Gymnocypris Przewalskii* (Cyprinidae) on the Tibetan plateau. Fish Shellfish Immunol. (2016) 50:34–42. doi: 10.1016/j.fsi.2016.01.012, PMID: 26774494

[2] Gou X Wang Z Li N Qiu F Xu Z Yan D. Whole-genome sequencing of six dog breeds from continuous altitudes reveals adaptation to high-altitude hypoxia. Genome Res. (2014) 24:1308–15. doi: 10.1101/gr.171876.113 24721644 PMC4120084

[3] Friedrich J Wiener P. Selection signatures for high-altitude adaptation in ruminants. Anim Genet. (2020) 51:157–65. doi: 10.1111/age.12900, PMID: 31943284

[4] Pritzkow C Williamson V Szota C Trouvé R Arndt SK. Phenotypic plasticity and genetic adaptation of functional traits influences intra-specific variation in hydraulic efficiency and safety. Tree Physiol. (2020) 40:215–29. doi: 10.1093/treephys/tpz121 31860729

[5] Ivy CM Scott GR. Control of breathing and the circulation in high-altitude mammals and birds. Comp Biochem Physiol A Mol Integr Physiol. (2015) 186:66–74. doi: 10.1016/j.cbpa.2014.10.009 25446936

[6] Ai H Yang B Li J Xie X Chen H Ren J. Population history and genomic signatures for high-altitude adaptation in Tibetan pigs. BMC Genomics. (2014) 15:834. doi: 10.1186/1471-2164-15-834, PMID: 25270331 PMC4197311

[7] Song S Yao N Yang M Liu X Dong K Zhao Q. Exome sequencing reveals genetic differentiation due to high-altitude adaptation in the Tibetan cashmere goat (*Capra Hircus*). BMC Genomics. (2016) 17:122. doi: 10.1186/s12864-016-2449-0, PMID: 26892324 PMC4758086

[8] Pham K Parikh K Heinrich EC. Hypoxia and inflammation: insights from high-altitude physiology. Front Physiol. (2021) 12:676782. doi: 10.3389/fphys.2021.676782, PMID: 34122145 PMC8188852

[9] Wang H Liu D Song P Jiang F Chi X Zhang T. Exposure to hypoxia causes stress erythropoiesis and downregulates immune response genes in spleen of mice. BMC Genomics. (2021) 22:413. doi: 10.1186/s12864-021-07731-x 34090336 PMC8178839

[10] Nazarov K Perik-Zavodskii R Perik-Zavodskaia O Alrhmoun S Volynets M Shevchenko J. Phenotypic alterations in erythroid nucleated cells of spleen and bone marrow in acute hypoxia. Cells. (2023) 12:2810. doi: 10.3390/cells12242810, PMID: 38132130 PMC10741844

[11] Yang S Zhang H Mao H Yan D Lu S Lian L. The local origin of the Tibetan pig and additional insights into the origin of Asian pigs. PLoS One. (2011) 6:e28215. doi: 10.1371/journal.pone.0028215, PMID: 22163285 PMC3233571

[12] Shang P Li WT Tan ZK Zhang J Dong SX Wang KJ. Population genetic analysis of ten geographically isolated Tibetan pig populations. Animals-Basel. (2020) 10:1297. doi: 10.3390/ani10081297 32751240 PMC7460208

[13] Zhang B Qiangba YZ Shang P Wang ZX Ma J Wang LY. A comprehensive Microrna expression profile related to hypoxia adaptation in the Tibetan pig. PLoS One. (2015) 10:e0143260. doi: 10.1371/journal.pone.0143260 26571238 PMC4646468

[14] Chen S Zhou Y Chen Y Gu J. Fastp: an ultra-fast all-in-one Fastq preprocessor. Bioinformatics. (2018) 34:i884–90. doi: 10.1093/bioinformatics/bty560, PMID: 30423086 PMC6129281

[15] Li H Durbin R. Fast and accurate short read alignment with burrows-wheeler transform. Bioinformatics. (2009) 25:1754–60. doi: 10.1093/bioinformatics/btp324, PMID: 19451168 PMC2705234

[16] Li H Handsaker B Wysoker A Fennell T Ruan J Homer N. The sequence alignment/map format and Samtools. Bioinformatics. (2009) 25:2078–9. doi: 10.1093/bioinformatics/btp352, PMID: 19505943 PMC2723002

[17] Tarasov A Vilella AJ Cuppen E Nijman IJ Prins P. Sambamba: fast processing of Ngs alignment formats. Bioinformatics. (2015) 31:2032–4. doi: 10.1093/bioinformatics/btv098, PMID: 25697820 PMC4765878

[18] McKenna A Hanna M Banks E Sivachenko A Cibulskis K Kernytsky A. The genome analysis toolkit: a Mapreduce framework for analyzing next-generation DNA sequencing data. Genome Res. (2010) 20:1297–303. doi: 10.1101/gr.107524.110, PMID: 20644199 PMC2928508

[19] Purcell S Neale B Todd-Brown K Thomas L Ferreira MA Bender D. Plink: a tool set for whole-genome association and population-based linkage analyses. Am J Hum Genet. (2007) 81:559–75. doi: 10.1086/519795 17701901 PMC1950838

[20] Danecek P Auton A Abecasis G Albers CA Banks E DePristo MA. The variant call format and Vcftools. Bioinformatics. (2011) 27:2156–8. doi: 10.1093/bioinformatics/btr330, PMID: 21653522 PMC3137218

[21] Gu H. Analysis of population genetic structure and selection signals in four Tibetan pig groups in China based on whole-genome resequencing. Master's thesis (2023).

[22] Patwardhan MN Wenger CD Davis ES Phanstiel DH. Bedtoolsr: an R package for genomic data analysis and manipulation. J Open Source Softw. (2019) 4:1742. doi: 10.21105/joss.01742, PMID: 31903447 PMC6941791

[23] Bapat A Schippel N Shi XJ Jasbi P Gu HW Kala M. Hypoxia promotes erythroid differentiation through the development of progenitors and Proerythroblasts. Exp Hematol. (2021) 97:32–46.e35. doi: 10.1016/j.exphem.2021.02.012, PMID: 33675821 PMC8102433

[24] Stonestreet BS Ocampo SS Oh W. Reductions in cardiac output in hypoxic young pigs: systemic and regional perfusion and oxygen metabolism. J Appl Physiol (1985). (1998) 85:874–82. doi: 10.1152/jappl.1998.85.3.874, PMID: 9729560

[25] Nys K Maes H Andrei G Snoeck R Garmyn M Agostinis P. Skin mild hypoxia enhances killing of Uvb-damaged keratinocytes through reactive oxygen species-mediated apoptosis requiring Noxa and Bim. Free Radic Biol Med. (2012) 52:1111–20. doi: 10.1016/j.freeradbiomed.2011.12.017, PMID: 22245094

[26] Karar J Maity A. Pi3k/Akt/Mtor Pathway in Angiogenesis. Front Mol Neurosci. (2011) 4:51. doi: 10.3389/fnmol.2011.00051, PMID: 22144946 PMC3228996

[27] Yang Y Yuan H Yang T Li Y Gao C Jiao T. The expression regulatory network in the lung tissue of Tibetan pigs provides insight into hypoxia-sensitive pathways in high-altitude hypoxia. Front Genet. (2021) 12:691592. doi: 10.3389/fgene.2021.691592, PMID: 34691141 PMC8529057

[28] Chen Y Meng X Wan R Cheng R Zhang G Zhang Q. Single-cell transcriptomic survey of cell diversity and functional changes in yak hearts at different altitude. Proteomics. (2023) 23:e2200345. doi: 10.1002/pmic.202200345, PMID: 36739517

[29] Zhang W Fan Z Han E Hou R Zhang L Galaverni M. Hypoxia adaptations in the Grey wolf (*Canis Lupus Chanco*) from Qinghai-Tibet plateau. PLoS Genet. (2014) 10:e1004466. doi: 10.1371/journal.pgen.1004466, PMID: 25078401 PMC4117439

[30] Hait NC Maiti A Xu P Qi Q Kawaguchi T Okano M. Regulation of hypoxia-inducible factor functions in the nucleus by Sphingosine-1-phosphate. FASEB J. (2020) 34:4293–310. doi: 10.1096/fj.201901734RR, PMID: 32017264 PMC10112293

[31] Owczarek A Gieczewska K Jarzyna R Jagielski AK Kiersztan A Gruza A. Hypoxia increases the rate of renal gluconeogenesis via hypoxia-inducible Factor-1-dependent activation of phosphoenolpyruvate Carboxykinase expression. Biochimie. (2020) 171-172:31–7. doi: 10.1016/j.biochi.2020.02.002, PMID: 32045650

[32] Park NR Shetye SS Bogush I Keene DR Tufa S Hudson DM. Reticulocalbin 3 is involved in postnatal tendon development by regulating collagen Fibrillogenesis and cellular maturation. Sci Rep-UK. (2021) 11:10868. doi: 10.1038/s41598-021-90258-8 PMC8149630 34035379

[33] Huang KY Liu ZY Xie ZL Li XR Zhang HX Chen Y. Higd2a silencing impairs hepatocellular carcinoma growth via inhibiting mitochondrial function and the Mapk/Erk pathway. J Transl Med. (2023) 21:253. doi: 10.1186/s12967-023-04105-7 37041638 PMC10091548

[34] Joshi HP Subramanian IV Schnettler EK Ghosh G Rupaimoole R Evans C. Dynamin 2 along with Microrna-199a reciprocally regulate hypoxia-inducible factors and ovarian Cancer metastasis. P Natl Acad Sci USA. (2014) 111:5331–6. doi: 10.1073/pnas.1317242111, PMID: 24706848 PMC3986124

[35] Galban S Gorospe M. Factors interacting with Hif-1alpha Mrna: novel therapeutic targets. Curr Pharm Des. (2009) 15:3853–60. doi: 10.2174/138161209789649376, PMID: 19671045 PMC2791462

[36] Ding X Zhou SJ Li M Cao C Wu PP Sun L. Upregulation of Srf is associated with hypoxic pulmonary hypertension by promoting viability of smooth muscle cells via increasing expression of Bcl-2. J Cell Biochem. (2017) 118:2731–8. doi: 10.1002/jcb.25922, PMID: 28176371

[37] Qi X Cui C Peng Y Zhang X Yang Z Zhong H. Genetic evidence of Paleolithic colonization and Neolithic expansion of modern humans on the Tibetan plateau. Mol Biol Evol. (2013) 30:1761–78. doi: 10.1093/molbev/mst093 23682168

[38] Lu D Lou H Yuan K Wang X Wang Y Zhang C. Ancestral origins and genetic history of Tibetan highlanders. Am J Hum Genet. (2016) 99:580–94. doi: 10.1016/j.ajhg.2016.07.002, PMID: 27569548 PMC5011065

[39] He G Wang M Zou X Chen P Wang Z Liu Y. Peopling history of the Tibetan plateau and multiple waves of admixture of Tibetans inferred from both ancient and modern genome-wide data. Front Genet. (2021) 12:725243. doi: 10.3389/fgene.2021.725243, PMID: 34650596 PMC8506211

[40] Kong X Dong X Yang S Qian J Yang J Jiang Q. Natural selection on Tmprss6 associated with the blunted erythropoiesis and improved blood viscosity in Tibetan pigs. Comp Biochem Physiol B Biochem Mol Biol. (2019) 233:11–22. doi: 10.1016/j.cbpb.2019.03.003, PMID: 30885835

[41] Ma YF Han XM Huang CP Zhong L Adeola AC Irwin DM. Population genomics analysis revealed origin and high-altitude adaptation of Tibetan pigs. Sci Rep. (2019) 9:11463. doi: 10.1038/s41598-019-47711-6 31391504 PMC6685962

[42] Wang T Guo YY Liu SW Zhang CX Cui TY Ding K. Acts on the Tgf-Β signaling pathway and contributes to high-altitude adaptation of Tibetan pigs. Front Genet. (2021):12. doi: 10.3389/fgene.2021.628192 PMC8082500 33936161

[43] Wang Z Li Q Chamba Y Zhang B Shang P Zhang H. Identification of genes related to growth and lipid deposition from transcriptome profiles of pig muscle tissue. PLoS One. (2015) 10:e0141138. doi: 10.1371/journal.pone.0141138, PMID: 26505482 PMC4624711

[44] Wang Z Li Q Chamba Y Zhang B Shang P Zhang H. Correction: identification of genes related to growth and lipid deposition from transcriptome profiles of pig muscle tissue. PLoS One. (2017) 12:e0172930. doi: 10.1371/journal.pone.0172930, PMID: 28225810 PMC5321414

[45] Wu F Sun H Lu S Gou X Yan D Xu Z. Genetic diversity and selection signatures within Diannan small-ear pigs revealed by next-generation sequencing. Front Genet. (2020) 11:733. doi: 10.3389/fgene.2020.00733, PMID: 32849777 PMC7406676

[46] Prabhakar NR Semenza GL. Adaptive and maladaptive cardiorespiratory responses to continuous and intermittent hypoxia mediated by hypoxia-inducible factors 1 and 2. Physiol Rev. (2012) 92:967–1003. doi: 10.1152/physrev.00030.2011 22811423 PMC3893888

[47] Wang B Zhang YB Zhang F Lin H Wang X Wan N. On the origin of Tibetans and their genetic basis in adapting high-altitude environments. PLoS One. (2011) 6:e17002. doi: 10.1371/journal.pone.0017002, PMID: 21386899 PMC3046130

[48] Wu DD Ding XD Wang S Wojcik JM Zhang Y Tokarska M. Pervasive introgression facilitated domestication and adaptation in the Bos species complex. Nat Ecol Evol. (2018) 2:1139–45. doi: 10.1038/s41559-018-0562-y, PMID: 29784979

[49] Wang Z Song B Yao J Li X Zhang Y Tang Z. Whole-genome analysis reveals distinct adaptation signatures to diverse environments in Chinese domestic pigs. J Anim Sci Biotechnol. (2024) 15:97. doi: 10.1186/s40104-024-01053-0 38982489 PMC11234542

[50] Tolonen JP Heikkila M Malinen M Lee HM Palvimo JJ Wei GH. A long hypoxia-inducible factor 3 isoform 2 is a transcription activator that regulates erythropoietin. Cell Mol Life Sci. (2020) 77:3627–42. doi: 10.1007/s00018-019-03387-9, PMID: 31768607 PMC7452874

[51] Suzuki N. Erythropoietin gene expression: developmental-stage specificity, cell-type specificity, and hypoxia inducibility. Tohoku J Exp Med. (2015) 235:233–40. doi: 10.1620/tjem.235.233, PMID: 25786542

[52] Liu Q Davidoff O Niss K Haase VH. Hypoxia-inducible factor regulates hepcidin via erythropoietin-induced erythropoiesis. J Clin Invest. (2012) 122:4635–44. doi: 10.1172/JCI63924 23114598 PMC3533545

[53] Haase VH. Regulation of erythropoiesis by hypoxia-inducible factors. Blood Rev. (2013) 27:41–53. doi: 10.1016/j.blre.2012.12.003, PMID: 23291219 PMC3731139

[54] Oba S Suzuki E Nishimatsu H Kumano S Hosoda C Homma Y. Renoprotective effect of erythropoietin in ischemia/reperfusion injury: possible roles of the Akt/endothelial nitric oxide synthase-dependent pathway. Int J Urol. (2012) 19:248–55. doi: 10.1111/j.1442-2042.2011.02920.x, PMID: 22126194

[55] Hafizi R Imeri F Stepanovska Tanturovska B Manaila R Schwalm S Trautmann S. Sphk1 and Sphk2 differentially regulate erythropoietin synthesis in mouse renal interstitial fibroblast-like cells. Int J Mol Sci. (2022) 23:5882. doi: 10.3390/ijms23115882, PMID: 35682566 PMC9180811

[56] Hetz C Zhang K Kaufman RJ. Mechanisms, regulation and functions of the unfolded protein response. Nat Rev Mol Cell Biol. (2020) 21:421–38. doi: 10.1038/s41580-020-0250-z, PMID: 32457508 PMC8867924

[57] Xiong Z Yuan C Shi J Xiong W Huang Y Xiao W. Restoring the epigenetically silenced Pck2 suppresses renal cell carcinoma progression and increases sensitivity to Sunitinib by promoting endoplasmic reticulum stress. Theranostics. (2020) 10:11444–61. doi: 10.7150/thno.48469, PMID: 33052225 PMC7546001

[58] Balaban RS. Cardiac energy metabolism homeostasis: role of cytosolic calcium. J Mol Cell Cardiol. (2002) 34:1259–71. doi: 10.1006/jmcc.2002.2082 12392982

[59] Cioffi DL Lowe K Alvarez DF Barry C Stevens T. Trping on the lung endothelium: calcium channels that regulate barrier function. Antioxid Redox Signal. (2009) 11:765–76. doi: 10.1089/ars.2008.2221, PMID: 18783312 PMC2850299

[60] Wobben R Husecken Y Lodewick C Gibbert K Fandrey J Winning S. Role of hypoxia inducible factor-1alpha for interferon synthesis in mouse dendritic cells. Biol Chem. (2013) 394:495–505. doi: 10.1515/hsz-2012-0320 23362200

[61] Parra-Izquierdo I Castanos-Mollor I Lopez J Gomez C San Roman JA Sanchez Crespo M. Lipopolysaccharide and interferon-gamma team up to activate Hif-1alpha via Stat1 in Normoxia and exhibit sex differences in human aortic valve interstitial cells. Biochim Biophys Acta Mol basis Dis. (2019) 1865:2168–79. doi: 10.1016/j.bbadis.2019.04.014, PMID: 31034990

[62] Tarnawski AS. Cellular and molecular mechanisms of gastrointestinal ulcer healing. Dig Dis Sci. (2005) 50:S24–33. doi: 10.1007/s10620-005-2803-6 16184417

[63] Franco CA Li Z. Srf in angiogenesis: branching the vascular system. Cell Adhes Migr. (2009) 3:264–7. doi: 10.4161/cam.3.3.8291, PMID: 19287204 PMC2712806

[64] Lopez-Maury L Marguerat S Bahler J. Tuning gene expression to changing environments: from rapid responses to evolutionary adaptation. Nat Rev Genet. (2008) 9:583–93. doi: 10.1038/nrg2398, PMID: 18591982

[65] Zhao P Li S He Z Zhao F Wang J Liu X. Physiology and proteomic basis of lung adaptation to high-altitude hypoxia in Tibetan sheep. Animals (Basel). (2022) 12:2134. doi: 10.3390/ani12162134, PMID: 36009723 PMC9405401

[66] Semenza GL. Hypoxia-inducible factors in physiology and medicine. Cell. (2012) 148:399–408. doi: 10.1016/j.cell.2012.01.021, PMID: 22304911 PMC3437543

[67] Kaufman RJ Malhotra JD. Calcium trafficking integrates endoplasmic reticulum function with mitochondrial bioenergetics. Biochim Biophys Acta. (2014) 1843:2233–9. doi: 10.1016/j.bbamcr.2014.03.022, PMID: 24690484 PMC4285153

[68] Chen Z Tian R She Z Cai J Li H. Role of oxidative stress in the pathogenesis of nonalcoholic fatty liver disease. Free Radic Biol Med. (2020) 152:116–41. doi: 10.1016/j.freeradbiomed.2020.02.025 32156524

[69] Battistini A. Interferon regulatory factors in hematopoietic cell differentiation and immune regulation. J Interf Cytokine Res. (2009) 29:765–80. doi: 10.1089/jir.2009.0030 19929577

