# Supplementary material for: Screening of functional genes for hypoxia adaptation in Tibetan pigs by combined genome resequencing and transcriptome analysis
Source: Front Vet Sci. 2024 Oct 21;11:1486258. doi: 10.3389/fvets.2024.1486258 (PMC11532106; doi:10.3389/fvets.2024.1486258)
Supplement: Supplementary file 1 [file Data_Sheet_1.docx]

Supplementary Material

1 Supplementary Figures and Tables

# 1.1 Supplementary Figures


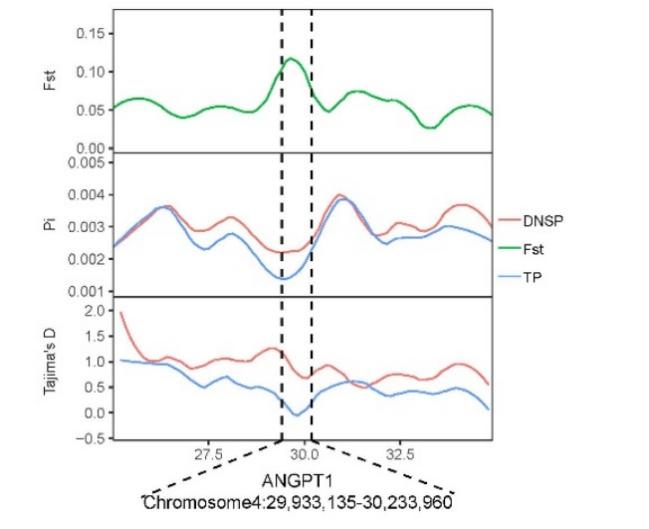

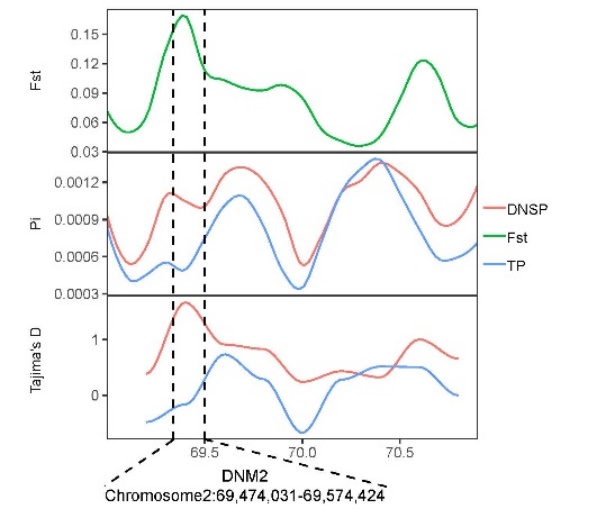


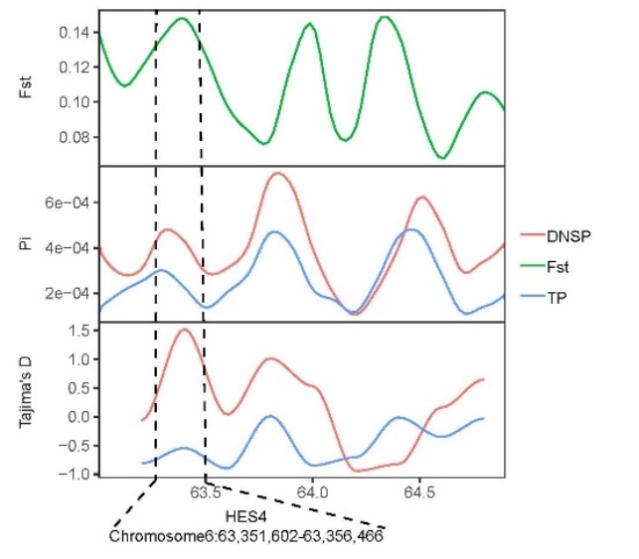

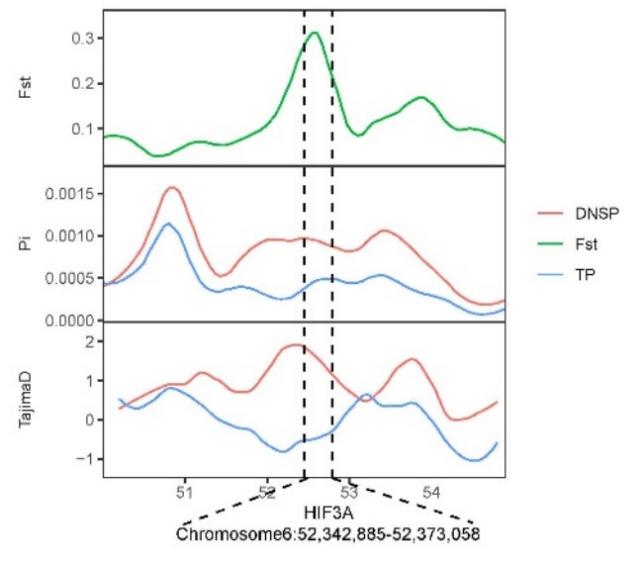


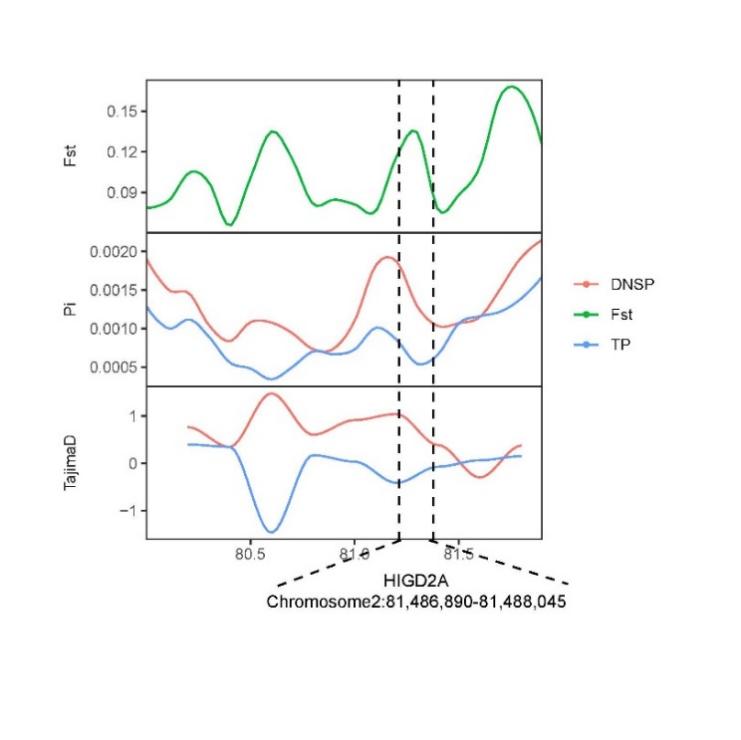

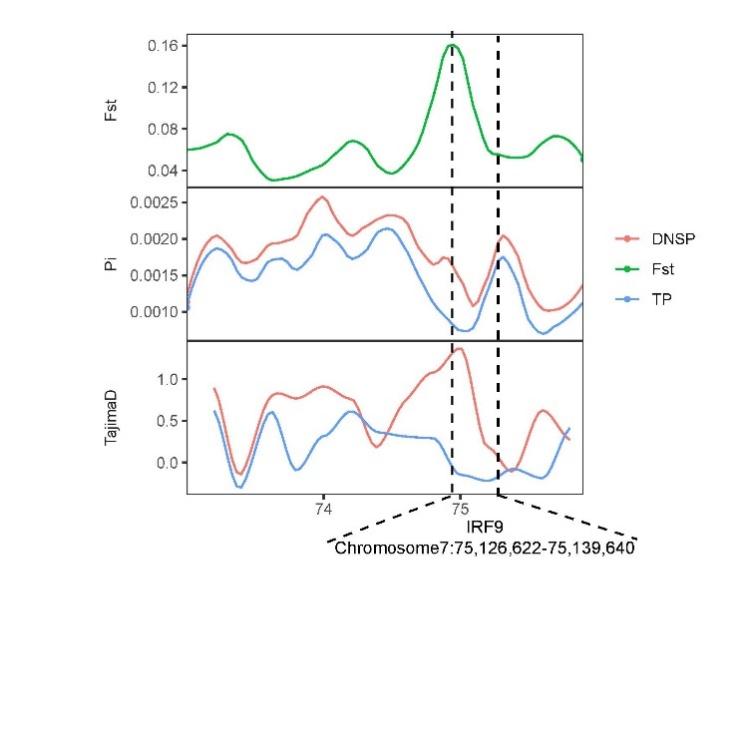


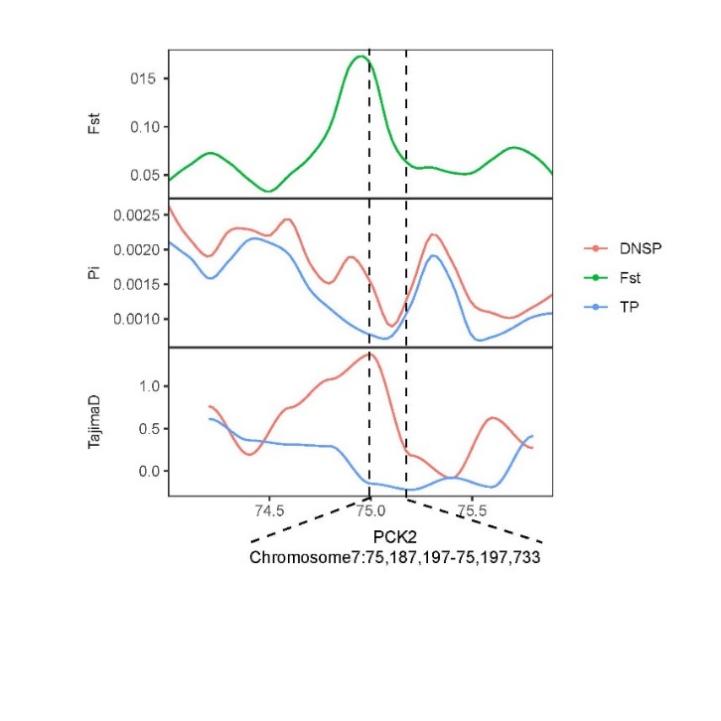

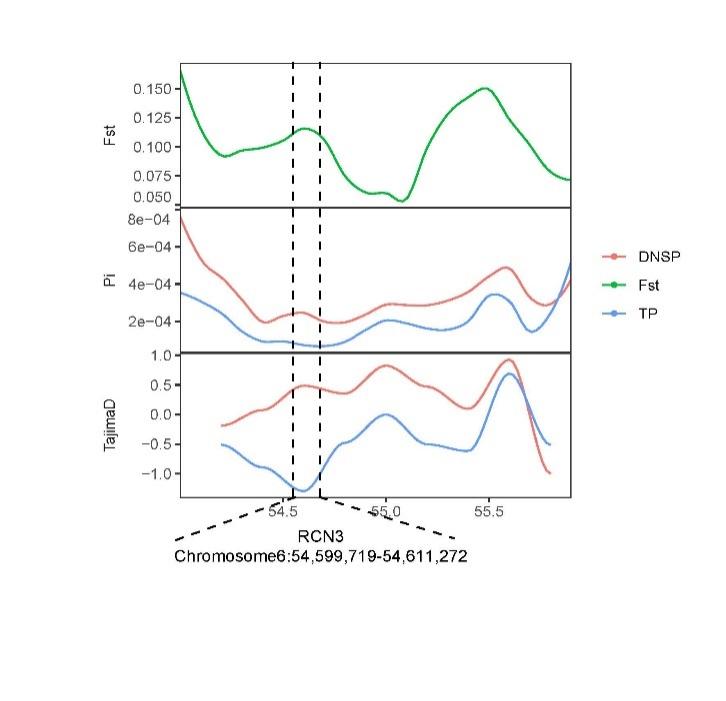

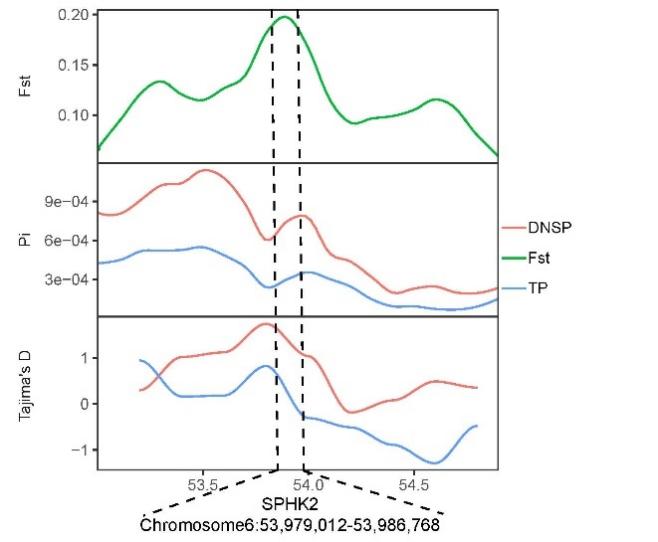

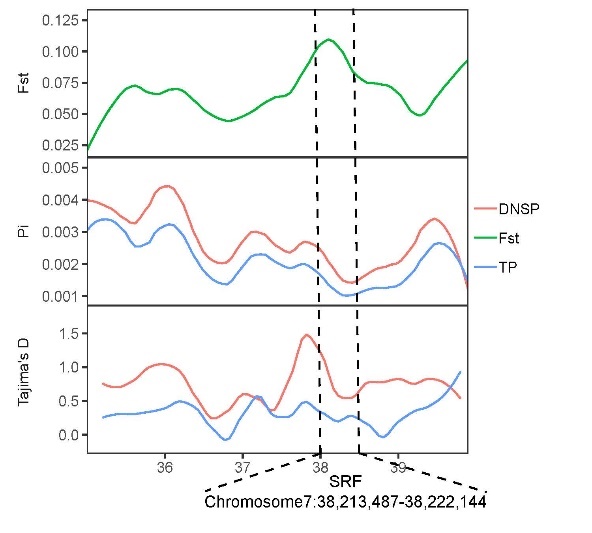


**Supplementary Figure 1.** Images of genomic Fst, nucleotide diversity and Tajima's D analysis(The selection signal analysis identified 10 genes—HES4, ANGPT1, HIF3A, SPHK2, PCK2, RCN3, HIGD2A, DNM2, IRF9, and SRF)

# 1.2 Supplementary Tables

**Supplementary Table 1.** Download data of Tibetan pig genome resequencing

| Species | SRR | Sample number |
| --- | --- | --- |
| Tibetan pig | SRR652257 | TP1 |
| Tibetan pig | SRR652258 | TP2 |
| Tibetan pig | SRR652259 | TP3 |
| Tibetan pig | SRR652260 | TP4 |
| Tibetan pig | SRR652261 | TP5 |
| Tibetan pig | SRR652262 | TP6 |
| Tibetan pig | SRR949672 | TP7 |
| Tibetan pig | SRR652264 | TP8 |
| Tibetan pig | SRR652265 | TP9 |
| Tibetan pig | SRR949674 | TP10 |
| Tibetan pig | SRR652267 | TP11 |
| Tibetan pig | SRR652268 | TP12 |
| Tibetan pig | SRR652269 | TP13 |
| Tibetan pig | SRR652270 | TP14 |
| Tibetan pig | SRR652302 | TP15 |
| Tibetan pig | SRR652303 | TP16 |
| Tibetan pig | SRR652304 | TP17 |
| Tibetan pig | SRR652305 | TP18 |
| Tibetan pig | SRR652306 | TP19 |
| Tibetan pig | SRR652307 | TP20 |
| Tibetan pig | SRR652327 | TP21 |
| Tibetan pig | SRR652340 | TP22 |
| Tibetan pig | SRR652341 | TP23 |
| Tibetan pig | SRR652342 | TP24 |
| Tibetan pig | SRR652343 | TP25 |
| Tibetan pig | SRR652344 | TP26 |
| Tibetan pig | SRR652345 | TP27 |
| Tibetan pig | SRR652346 | TP28 |
| Tibetan pig | SRR652347 | TP29 |
| Tibetan pig | SRR949678 | TP30 |

**Supplementary Table 2.** Genome resequencing Quality control and comparison of data

| Sample ID | raw_reads | clean_reads | GC (%) | Q20 (%) | Q30 (%) | | Mapping ratio(%) |
| --- | --- | --- | --- | --- | --- | --- | --- |
| DNSP1 | 431457830 | 431318532 | 44.12 | 96.80 | 92.05 | 99.55 | |
| DNSP2 | 311237916 | 311139070 | 42.74 | 96.33 | 90.77 | 99.58 | |
| DNSP3 | 398479562 | 398348996 | 43.96 | 96.86 | 92.19 | 99.27 | |
| DNSP4 | 481724234 | 481568306 | 43.90 | 96.72 | 91.87 | 99.51 | |
| DNSP5 | 316932390 | 316835260 | 42.59 | 95.91 | 89.69 | 99.48 | |
| DNSP6 | 575274998 | 575089302 | 43.78 | 96.77 | 91.97 | 99.61 | |
| DNSP7 | 383096460 | 382972964 | 43.83 | 96.76 | 91.91 | 99.53 | |
| DNSP8 | 496984308 | 496825932 | 44.02 | 96.59 | 91.56 | 99.53 | |
| DNSP9 | 311907718 | 311806038 | 44.16 | 96.78 | 91.99 | 99.57 | |
| DNSP10 | 311273572 | 311176144 | 42.63 | 96.09 | 90.16 | 99.59 | |
| DNSP13 | 322468728 | 322370498 | 42.06 | 95.85 | 89.55 | 99.62 | |
| DNSP14 | 297102878 | 297009980 | 42.69 | 96.44 | 91.16 | 99.62 | |
| DNSP16 | 281076670 | 280985338 | 42.65 | 96.67 | 91.69 | 99.65 | |
| DNSP17 | 269614318 | 269528526 | 43.01 | 96.45 | 91.20 | 99.52 | |
| DNSP18 | 306778252 | 306680700 | 43.11 | 96.56 | 91.41 | 99.61 | |
| DNSP19 | 321559018 | 321460492 | 42.65 | 95.90 | 89.61 | 99.61 | |
| DNSP20 | 307649730 | 307552936 | 42.55 | 96.17 | 90.35 | 99.54 | |
| DNSP21 | 317374314 | 317273226 | 42.52 | 96.23 | 90.48 | 99.64 | |
| DNSP22 | 317757646 | 317609098 | 42.76 | 96.12 | 89.71 | 99.63 | |
| DNSP23 | 320448542 | 320299382 | 42.80 | 96.02 | 89.46 | 99.65 | |
| DNSP24 | 302347410 | 302225746 | 42.49 | 95.70 | 88.73 | 99.64 | |
| DNSP25 | 321679126 | 321555436 | 42.89 | 94.98 | 87.22 | 99.64 | |
| DNSP26 | 295765184 | 295618406 | 42.76 | 96.17 | 89.85 | 99.63 | |
| DNSP27 | 326604968 | 326448644 | 43.62 | 96.25 | 90.01 | 94.82 | |
| DNSP30 | 326500540 | 326360428 | 42.87 | 95.74 | 88.86 | 99.59 | |
| DNSP31 | 301047220 | 300897658 | 43.02 | 96.19 | 89.86 | 99.65 | |
| DNSP32 | 339252562 | 339110690 | 42.56 | 95.43 | 88.10 | 99.58 | |
| DNSP34 | 344732816 | 344584378 | 42.54 | 95.91 | 89.20 | 99.64 | |
| DNSP35 | 328421744 | 328268722 | 42.79 | 96.12 | 89.71 | 99.66 | |
| DNSP36 | 318052170 | 317897710 | 42.71 | 96.11 | 89.69 | 99.66 | |
| TP1 | 118793070 | 116646110 | 40.36 | 98.15 | 94.62 | 99.68 | |
| TP2 | 121527990 | 118261440 | 40.94 | 96.82 | 90.61 | 99.54 | |
| TP3 | 106355728 | 103498568 | 40.15 | 96.88 | 90.82 | 99.55 | |
| TP4 | 142929146 | 139335254 | 40.68 | 96.98 | 91.05 | 99.55 | |
| TP5 | 142269914 | 137695652 | 40.87 | 96.58 | 90.16 | 99.54 | |
| TP6 | 160484226 | 155768040 | 39.87 | 96.69 | 90.39 | 99.63 | |
| TP7 | 326407388 | 322557382 | 41.10 | 96.61 | 90.71 | 99.54 | |
| TP8 | 116672738 | 114266336 | 40.67 | 97.92 | 93.94 | 99.63 | |
| TP9 | 176129024 | 171626046 | 39.75 | 97.20 | 91.78 | 99.62 | |
| TP10 | 73277744 | 72692134 | 41.24 | 98.48 | 95.14 | 99.16 | |
| TP11 | 97264596 | 94955418 | 38.76 | 97.73 | 93.40 | 99.7 | |
| TP12 | 190398892 | 185281644 | 40.06 | 97.13 | 91.61 | 99.63 | |
| TP13 | 134002166 | 130366350 | 39.21 | 97.25 | 91.98 | 99.68 | |
| TP14 | 121504770 | 117527512 | 39.15 | 96.53 | 90.04 | 99.69 | |
| TP15 | 178695304 | 171742134 | 39.13 | 96.04 | 89.23 | 99.67 | |
| TP16 | 147019082 | 141089522 | 38.67 | 96.23 | 89.85 | 99.69 | |
| TP17 | 114855652 | 111819930 | 38.63 | 97.18 | 91.77 | 99.7 | |
| TP18 | 150496378 | 146499658 | 38.63 | 97.18 | 91.73 | 99.48 | |
| TP19 | 124056918 | 120381886 | 39.38 | 96.92 | 91.12 | 99.65 | |
| TP20 | 148672844 | 142657502 | 38.99 | 96.25 | 89.93 | 99.68 | |
| TP21 | 155627930 | 149462064 | 39.44 | 96.35 | 90.15 | 99.67 | |
| TP22 | 129444282 | 122470948 | 38.60 | 95.61 | 88.58 | 99.69 | |
| TP23 | 123617310 | 121372652 | 40.73 | 98.13 | 94.58 | 99.67 | |
| TP24 | 115607598 | 113166034 | 39.90 | 97.94 | 94.15 | 99.64 | |
| TP25 | 114658264 | 109580964 | 39.86 | 95.70 | 88.44 | 99.69 | |
| TP26 | 185832898 | 178318762 | 39.67 | 95.95 | 88.95 | 99.59 | |
| TP27 | 144564178 | 138445636 | 39.69 | 95.89 | 88.82 | 99.61 | |
| TP28 | 185198962 | 173748294 | 38.95 | 94.15 | 86.00 | 99.68 | |
| TP29 | 150856630 | 139853820 | 38.89 | 94.49 | 86.75 | 99.58 | |
| TP30 | 315317626 | 294333008 | 40.15 | 94.52 | 87.73 | 99.54 | |

**Supplementary Table 3.** Quality control and comparison of transcriptome data

| Sample_ID | Q20(%) | Q30(%) | GC content(%) | Overall alignment rate(%) |
| --- | --- | --- | --- | --- |
| TP1_heart | 98.51 | 95.37 | 47.64 | 95.89 |
| TP2_heart | 98.51 | 95.37 | 47.94 | 96.25 |
| TP3_heart | 98.56 | 95.53 | 47.99 | 96.19 |
| DNSP1_heart | 98.69 | 95.88 | 45.11 | 95.9 |
| DNSP2_heart | 98.51 | 95.27 | 46.46 | 95.86 |
| DNSP3_heart | 98.61 | 95.64 | 47.35 | 95.71 |
| TP1_liver | 98.61 | 95.55 | 50.45 | 96.19 |
| TP2_liver | 98.52 | 95.40 | 51.01 | 96.83 |
| TP3_liver | 98.58 | 95.60 | 50.45 | 96.75 |
| DNSP1_liver | 98.61 | 95.58 | 49.74 | 96.78 |
| DNSP2_liver | 98.61 | 95.75 | 45.71 | 95.47 |
| DNSP3_liver | 98.46 | 95.37 | 46.91 | 95.21 |
| TP1_spleen | 96.58 | 92.88 | 58.11 | 96.22 |
| TP2_spleen | 97.10 | 93.97 | 56.83 | 96.95 |
| TP3_spleen | 97.47 | 94.71 | 56.99 | 97.32 |
| DNSP1_spleen | 96.39 | 92.57 | 56.03 | 97.58 |
| DNSP2_spleen | 96.67 | 93.11 | 55.35 | 97.02 |
| DNSP3_spleen | 97.23 | 94.23 | 56.56 | 97.21 |
| TP1_lung | 96.93 | 93.76 | 53.19 | 95.81 |
| TP2_lung | 96.54 | 92.91 | 54.58 | 93.43 |
| TP3_lung | 97.44 | 94.59 | 56.25 | 96.87 |
| DNSP1_lung | 97.12 | 94.01 | 54.27 | 95.81 |
| DNSP2_lung | 97.86 | 95.53 | 54.52 | 97.33 |
| DNSP3_lung | 97.17 | 94.11 | 54.76 | 97.71 |
| TP1_bone marrow | 97.57 | 94.86 | 56.03 | 98.03 |
| TP2_bone marrow | 97.32 | 94.45 | 55.08 | 97.37 |
| TP3_bone marrow | 97.60 | 95.05 | 56.96 | 97.42 |
| DNSP1_bone marrow | 97.45 | 94.68 | 55.53 | 97.28 |
| DNSP2_bone marrow | 97.51 | 94.73 | 57.49 | 98.12 |
| DNSP3_bone marrow | 98.06 | 95.86 | 58.17 | 98.25 |

**Supplementary Table 4.** The sequences of the internal primers used for RT-qPCR

| Gene | Primer | Tissue |
| --- | --- | --- |
| HIGD2A | F: GAAGTTTATTCGCAAGACCCG  R: GCCAGACCCACCAAGATGAC | Liver |
| HIF3A | F: AGTCCTCTTCCGGCTGATCT  R: GTCTCCACTGCATCCAGGTC | Heart |
| PCK2 | F: CAGCCGAACACAAAGGGAAG  R: TCCATGCTCAGCCAGTGTTC | Liver |
| RCN3 | F: GTCACTTCTACTGCTGCTGTTGCT  R: TTCCTCTGGGCTGAGTTGGTC | Lung |
| IRF9 | F: AGTCTCCGGAAGGTAGGGTT  R: CCAGGACACCTCTCTTGAGC | Bone marrow |
